# SNAI1 promotes the development of HCC through the enhancement of proliferation and inhibition of apoptosis

**DOI:** 10.1002/2211-5463.12043

**Published:** 2016-03-10

**Authors:** Jianni Qi, Tao Li, Hongjun Bian, Feifei Li, Ying Ju, Shanshan Gao, Jingran Su, Wanhua Ren, Chengyong Qin

**Affiliations:** ^1^ Central LaboratoryShandong Provincial Hospital Affiliated to Shandong UniversityJinanShandongChina; ^2^ Department of GastroenterologyShandong Provincial Hospital Affiliated to Shandong UniversityJinanShandongChina; ^3^ Department of EmergencyShandong Provincial Hospital Affiliated to Shandong UniversityJinanShandongChina; ^4^ Department of Clinical LaboratoryShandong Provincial Hospital Affiliated to Shandong UniversityJinanShandongChina

**Keywords:** Bcl‐2, Cyclin D1, hepatocellular carcinoma, RNAi, SNAI1

## Abstract

SNAI1, a zinc‐finger transcription factor, plays an important role in the induction of epithelial–mesenchymal transition (EMT) in various cancers. However, the possible functions of SNAI1 in the proliferation and apoptosis of hepatocellular carcinoma have not been clearly identified. In this study, we investigated the effects and mechanisms of SNAI1 in the proliferation and apoptosis of hepatocellular carcinoma using clinical samples and cell lines. We found that SNAI1 is highly expressed in the tissues of liver cancer compared with adjacent nontumor tissues. SNAI1 is also highly expressed in the hepatoma cell lines HepG2, SMMC‐7721, and BEL‐7402 compared with the human normal liver cell line L02. We also observed that SNAI1 expression was correlated with distal metastasis, incomplete tumor capsule formation, and histological differentiation in hepatocellular carcinoma (HCC). Moreover, we demonstrated that knockdown of SNAI1 via lentiviral vectors of RNAi against SNAI inhibited cell proliferation by inducing G1 arrest, which was accompanied by the downregulation of cyclin D1 but not that of cyclin A. In addition, knockdown of SNAI1 promoted apoptosis by decreasing the expression of Bcl‐2. In conclusion, our findings revealed that SNAI1 is involved in the development of hepatocellular carcinoma via regulating the growth and apoptosis of tumor cells.

**Abbreviations**
DMEM: Dulbecco's modified Eagle's medium
EGFP: enhanced green fluorescent protein
EMT: epithelial–mesenchymal transition
HCC: hepatocellular carcinoma
ROC: receiver operating characteristic

Hepatocellular carcinoma (HCC) is a major worldwide health problem, particularly in China. It is the third leading cause of cancer‐related death in the world and the second leading cause of cancer‐related death in China 1, 2. To date, therapies for HCC, including surgical resection and local ablation, are insufficient, and the long‐term prognosis of HCC is extremely poor 3. Hence, HCC remains an intractable disease. Therefore, dissecting the molecular mechanisms involved in hepatoma carcinoma cell survival and growth is essential for the development of targeted therapies to reduce patient mortality.

The transition of epithelial cells to a mesenchymal phenotype, named epithelial‐to‐mesenchymal transition (EMT), has been recognized during the progression of various carcinomas, including HCC 4, 5. It has been implicated that EMT is one of the critical mechanisms to promote tumorigenesis through the disposal of their differentiated features, including cell‐to‐cell adhesion and apical‐basal polarity, and gaining of mesenchymal characteristics such as growth, proliferation, migration, invasiveness, and increased apoptotic resistance 6. SNAI1 is a zinc‐finger transcriptional repressor that has a highly conserved C‐terminal domain, including four to six C2H2‐type zinc fingers that bind to the E‐box 7. Compelling evidence has demonstrated that SNAI1 plays a key role in the induction of EMT 8, 9. Our previous study has observed by western blotting that SNAI1 is overexpressed in tissue specimens of HCC, and it is positively correlated with HCC progression and a worse outcome by receiver operating characteristic (ROC) and survival analysis 10. However, the potential mechanisms underlying the regulation of HCC remain incompletely understood.

In this study, we constructed lentiviral‐mediated RNAi vectors against SNAI1 and transfected them into HepG2 cell lines *in vivo* and *in vitro*. We found that the growth and migration of HepG2 cell lines were inhibited, and their apoptosis was enhanced after the silencing of SNAI1 expression *in vitro*. Mechanistically, we found that knockdown of SNAI1 decreased the expression of Cyclin D1 but increased the expression of Bcl‐2. Furthermore, our data demonstrated that this effect existed *in vivo*.

## Materials and methods

### Cell culture

The human hepatoma cell lines HepG2, SMMC‐7721, and BEL‐7402 and human live cell line L02 were obtained from the Shanghai Cell Collection (Chinese Academy of Sciences, Beijing, China). The HepG2 and SMMC‐7721 cell lines were cultured in Dulbecco's modified Eagle's medium supplemented with high glucose (DMEM; HyClone, Logan, UT, USA) containing 10% (vol/vol) fetal bovine serum (FBS; Gibco, Grand Island, NY, USA), and the BEL‐7402 and L02 cell lines were cultured in RPMI1640 medium supplemented with 10% (vol/vol) fetal bovine serum, 100 U·mL^−1^ penicillin, and 100 μg·mL^−1^ streptomycin. All of the cell lines were maintained at 37 °C in a humidified incubator with 5% CO_2_.

### Transfection

All of the lentiviral vectors expressed enhanced green fluorescent protein (EGFP), which allowed the measurement of their infection efficiency in transfected cells. LV‐SNAI1‐RNAi #1, #2, #3 and scrambled control LV‐RNAi were obtained from GENECHEM (Shanghai, China). The LV‐SNAI1‐RNAi #1 sequence was 5′‐CCACTCAGATGTCAAGAAGTA‐3′. The LV‐SNAI1‐RNAi #2 sequence was 5′‐CCAAGGATCTCCAGGCTCGAA‐3′. The LV‐SNAI1‐RNAi #3 sequence was 5′‐GCAGGACTCTAATCCAGAGTT‐3′. The scrambled control LV‐ RNAi sequence was 5′‐TTCTCCGAACGTGTCACGT‐3′. These lentiviral vectors were transfected into HepG2 cells with a multiplicity of infection (MOI) of 20 in the presence of enhancer solution and polybrene (5 μg·mL^−1^). After 14–16 h, the supernatant, including Enhanced Infection Solution, was removed, and fresh, complete medium was added. The cells were cultured for another 24, 48, 96, and/or 120 h before subsequent experiments.

### Real‐time PCR

Total RNA was extracted using TRIzol reagent (Invitrogen, Carlsbad, CA, USA) according to the manufacturer's instructions, and reverse transcription was performed using an RT‐PCR kit (TaKaRa, Kusatsu, Shiga, Japan). The expression of SNAI1 was quantified using real‐time quantitative PCR using SYBR Premix Ex Tap^™^ (TaKaRa) with β‐actin as an internal normalized reference. The sequences of primers were 5′‐ ACAAGCACCAAGAGTCCG‐3′(forward) and 5′‐CCCTCCCTCCACAGAAAT‐3′ (reverse) for SNAI1 and 5′‐TGTTACCAACTGGGACGACA‐3′(forward) and 5′‐CTGGGTCATCTTTTCACGGT‐3′ (reverse) for β‐actin. Quantitative polymerase chain reaction (PCR) was performed according to the following steps: 95 °C for 30 s, followed by 45 cycles of 95 °C for 5 s, 58 °C for 20 s, 72 °C for 20 s, and 65 °C for 20 s, using the LightCycler Real‐time PCR System (Roche Diagnostics, Indianapolis, IN, USA) as described previously 11.

### Western blotting

For western blot analysis, cells were lysed with the CelLytic^™^ Cell Lysis Reagent (Sigma, Saint Louis, MO, USA) supplemented with a protease inhibitor ‘cocktail’, the protein concentrations in the extracts were measured using the bicinchoninic acid assay (Pierce, Rockford, IL, USA), and then, the volumes were made equal using the extraction reagent. Equal amounts of extracts were separated by SDS/PAGE, and then, they were transferred onto polyvinylidene fluoride membranes for immunoblot analysis as described previously 12, 13. Rabbit polyclonal antibody to SNAI1 (ab‐17732; Abcam, Cambridge, MA, USA), mouse mAb to Cyclin D1 (ab6152; Abcam, Cambridge, MA, USA), mouse mAb to Cyclin A (4656; Cell Signaling Technology, Boston, MA, USA), rabbit mAb to Bcl‐2 (2870; Cell Signaling Technology), rabbit mAb to cytochrome c (4280; Cell Signaling Technology), mouse mAb to anti‐Caspase‐3 (9668; Cell Signaling Technology), mouse mAb to N‐Cadherin (14215; Cell Signaling Technology), rabbit mAb to E‐Cadherin (3195; Cell Signaling Technology), mouse mAb to β‐actin (TA‐09; ZSGB‐BIO, Beijing, China), and the corresponding HRP‐conjugated secondary antibody (sc‐2004 and sc‐2005; Santa Cruz Biotechnology, Dallas, TX, USA) were used for immunoblot analysis.

### CCK‐8 assay

Cell proliferation was evaluated using the Cell Counting Kit‐8 (CCK‐8; Beyotime, Shanghai, China). Cells were seeded at a concentration of 2.5 × 10^3^/well in 96‐well plates and then were transfected with scrambled control LV‐RNAi and LV‐SNAI1‐RNAi. After 0, 24, 48, 72, and 96 h, 10 μL of CCK‐8 solution was added to each well and incubated at 37 °C for 1 h. At the end of the incubation, the optical density was read at 450 nm using a plate reader (SoftMax Pro; Molecular Devices Corporation Sunnyvale, Sunnyvale, CA, USA). The average values were determined from different wells.

### Flow cytometry analysis

For cell cycle analysis, cells were collected 72 h after transfection with scrambled control or three LV‐SNAI1‐RNA is and then were fixed with cold 75% ethanol for 2 h at 4 °C. The fixed cells were centrifuged and then washed 3 times with PBS. Next, the cells were resuspended in 1 mL of PBS, and 100 μL of 500 μg·mL^−1^ RNaseA was added followed by incubation of the cells at 37 °C for 30 min. The cells were stained with propidium iodide (Sigma) to a final concentration of 5 μg·mL^−1^ containing 0.2% Triton X‐100 and incubated at 4 °C for 30 min in the dark. The cells were then subjected to flow cytometry (Beckman Coulter, Fullerton, CA, USA) for cell cycle analysis. The apoptosis assay was performed on HepG2 cells after 96 h of transfection with either the control or three LV‐SNAI1‐RNAis using the Annexin V‐PE/7‐ADD Apoptosis Detection Kit (KeyGen Biotech, Nanjing, China) according to the manufacturer's protocol, and then the cells were analyzed by flow cytometry.

### Transwell assay

The CytoSelect^™^ Boyden Chamber was obtained from CellBiolabs (San Diego, CA, USA). Cells transfected for 48 or 72 h were collected and washed with PBS. These cells were resuspended in 200 μL of DMEM without FBS and seeded to the upper chamber at a concentration of 1.5 × 10^5^ cells per well. Next, 600 μL of DMEM with 10% FBS was added to the lower chamber. After incubation for 18 h, the cells that traversed and spread on the lower surface of the membrane were fixed with 4% paraformaldehyde, and nonmigrated cells on the upper surface of the filter were removed with a cotton swab and stained with hematoxylin. The average number of cells per field was counted by × 10 objective magnification from five microscopic fields.

### *In vivo* tumor growth assay

Male BALB/c nude mice at 3–4 weeks of age were purchased from the Animal Research Committee of the Institute of Biology and Cell Biology (Shanghai, China) and housed in a specific pathogen‐free environment. The animal room was kept at 20–22 °C under a 12‐h light/dark cycle. HepG2 cells (1 × 10^7^) were subcutaneously transplanted into the posterior flank of nude mice. After reaching a diameter of 0.5 cm, these mice were randomly divided into two groups, and 2 × 10^6^ TU of control LV‐ RNAi or LV‐SNAI1‐RNAi #3 vectors, respectively, were injected into each mouse every 2 days for a total of 6–8 injections. The tumor size was monitored every 2 days and calculated as V (mm^3^) = width^2^ (mm^2^) × length (mm)/2 as described previously 14. Mice were sacrificed 3–4 days after the final injection, and the tumors were isolated and weighed. Animal experiments were repeated at least three times, and three mice were included in each group. All animal studies were performed in accordance with the National Institutes of Health Guide for the Care and Use of Laboratory Animals, with the approval of the Animal Research Committee of the Medical School of Shandong University, Jinan, Shandong Province, China.

### Immunohistochemistry analysis

Paraffin‐embedded tissue sections, obtained from the Department of Pathology of Shandong Provincial Hospital affiliated with Shandong University were deparaffinized in xylene and rehydrated through graded alcohol solutions. Antigen retrieval was performed for 15 min at 98 °C in citrate buffer (pH 6.0) in a water bath. Endogenous peroxidases were inactivated by immersing the sections in 0.3% H_2_O_2_ for 30 min at 37 °C. The sections were incubated at 4 °C with rabbit polyclonal antibody SNAI1 (dilution 1 : 50) overnight in a humidified chamber and then incubated with SABC (SA1022; Boster, WuHan, China) for 40 min at 37 °C. Staining results were viewed under a light microscope (Olympus, Leeds Precision Instruments, Minneapolis, MN, USA), and pictures were taken with an imaging program. Written informed consent was acquired from each patient for this study. The study methodologies conformed to the standards set by the Declaration of Helsinki. The research protocol and consent program were approved by the Shandong Provincial Hospital Affiliated with Shandong University Medical Institutional Ethical Committee.

### Statistical analysis

The spss 16.0 statistical software (Chicago, IL, USA) was used for all data analyses. To evaluate significant differences between the groups, Student's *t* test, the Mann–Whitney *U*‐test or one‐way analysis of variance (anova) were applied. To determine significant differences between different treatments or time points, two‐way anova was used. The Chi‐squared test was applied to analyze the statistical correlation between the clinical parameters of HCC and SNAI1 staining levels in tissue sections. In all cases, a *P* value less than 0.05 was considered to be statistically significant.

## Results

### SNAI1 expression is upregulated in HCC tissue and is correlated with certain clinical parameters

**Figure 1:**
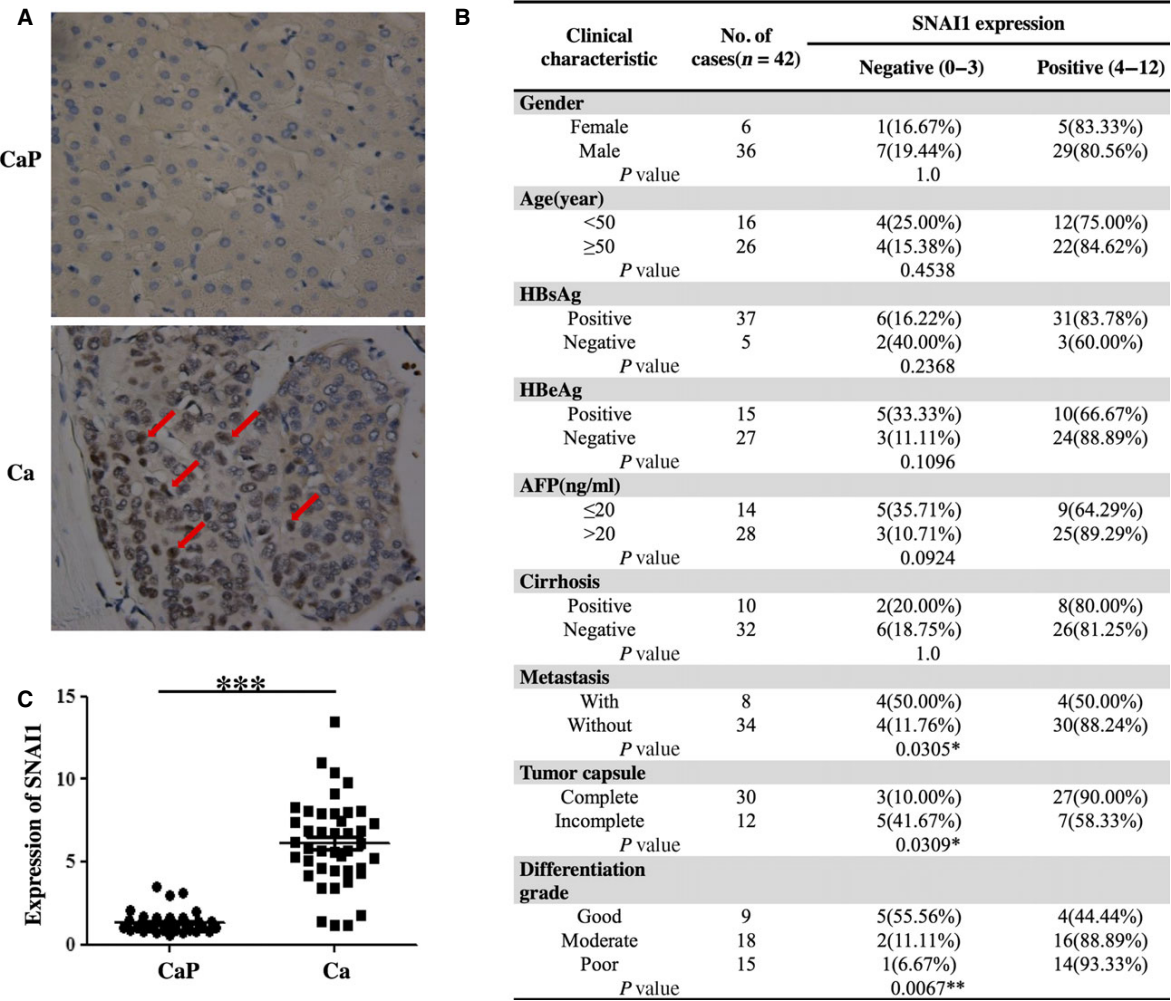
SNAI1 expression is upregulated in HCC tissue and is correlated with certain clinical parameters. In the clinic, samples of HCC and adjacent benign tissues were collected, and SNAI1 expression was detected by immunohistochemistry (A: CaP, adjacent noncancer; Ca, cancer. Original magnification, × 200) and real‐time PCR (C). The immunoreactive score of SNAI1 expression and different clinical parameters were statistically analyzed in HCC tissues (B). ***P < 0.001.

**Figure 2:**
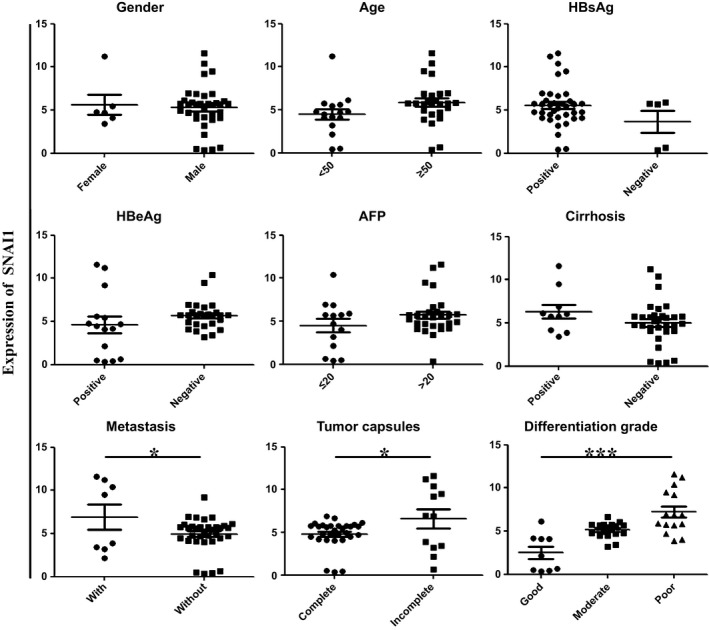
SNAI1 expression is correlated with distal metastasis, incomplete tumor capsule formation, and histological differentiation. The levels of SNAI1 expression were well measured using real‐time PCR. The correlation of SNAI1 expression and clinical features, included cirrhosis, metastasis, tumor capsule formation and histological grade, was analyzed. *P < 0.05, ***P < 0.001.

To verify the potential role of SNAI1 in HCC, we first detected its expression by immunohistochemistry and real‐time PCR in 42 pairs of HCC and adjacent benign tissues. We found that the expression of SNAI1 was significantly increased compared with that of adjacent nontumor tissues (Fig. 1A,C). Increased expression of SNAI1 was observed in 80.95% of HCC (34 of 42 cases). The above results were consistent with those of our previous reports 10. Meanwhile, we analyzed the correlation with SNAI1 expression and clinical features of tumor progression and disease prognosis. As shown in Figs 1B and 2, the expression of SNAI1 was significantly higher in patients with distal metastasis than in patients without distal metastasis. In addition, patients with incomplete tumor capsule formation had higher levels of SNAI1 expression than patients with complete tumor capsule formation. Furthermore, patients with a poorly differentiated grade had remarkably higher SNAI1 expression than patients with a good differentiated grade. However, no significant differences were observed for the levels of SNAI1 expression regarding gender, age, HBsAg, HBeAg, AFP and cirrhosis. Taken together, these data indicate that a higher expression of SNAI1 may accelerate tumor invasion and metastasis, functions that may be correlated with a poor prognosis.

### Identification of an efficient RNAi sequence against SNAI1

**Figure 3:**
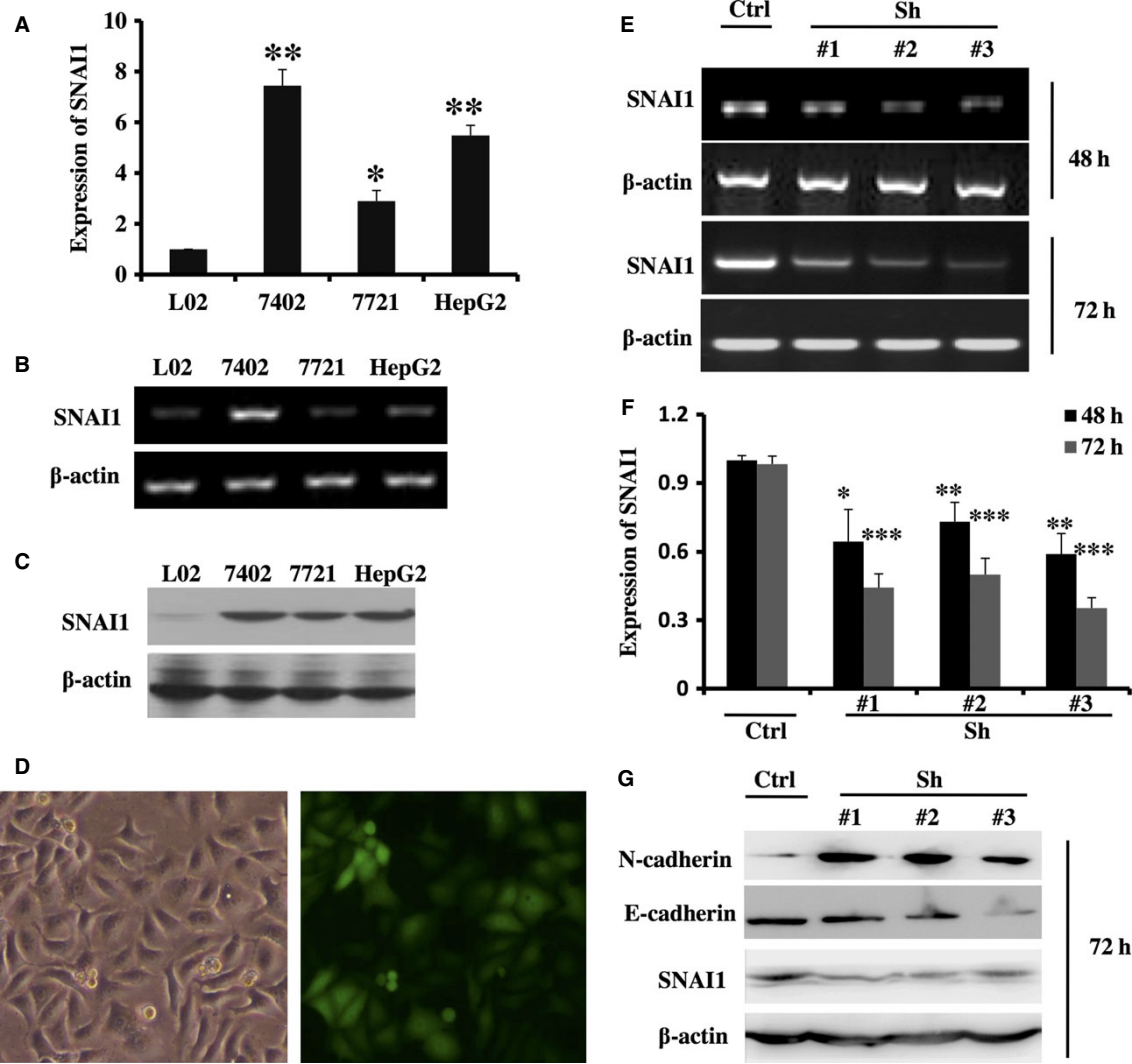
SANI1 expression is effectively decreased by three lentiviral vectors expressing RNAi against SNAI1. Nonquantitative and quantitative PCR (A, B) and western blot (C) assay measured the SNAI1 expression in L02, BEL‐7402, SMMC‐7721 and HepG2 cell lines. β‐actin confirmed the equal level of mRNA or protein in each sample. After scrambled control lentiviral vectors were transfected into HepG2 cells with a MOI of 20 for 48 h, the transfection efficiency was observed with fluorescence microscope (D). After lentiviral vectors expressing RNAi against SNAI1 or scrambled control vectors were transfected into HepG2 cells with a MOI of 20 for 48 or 72 h, HepG2 cells were collected with TRIzol reagent and nonquantitative (E), quantitative PCR (F) and western blotting (G) were performed to examine the expression of SNAI1. Western blotting (G) examined the expression of E‐cadherin and N‐cadherin. β‐actin is an internal normalized reference. Similar results were obtained in three independent experiments. *P < 0.05, **P < 0.01, ***P < 0.001.

To determine the effect of upregulated SNAI1 in HCC, three lentiviral vectors expressing RNAi against SNAI1 (LV‐SNAI1‐RNAi #1, #2 and #3) were constructed. First, we detected the expression of SNAI1 in the HCC cell lines BEL‐7402, SMMC‐7721 and HepG2. As shown in Fig. 3A,B,C, SNAI1 expression in the above three HCC cell lines was markedly upregulated compared with that in the human normal liver cell line L02. Second, we transfected three lentiviral vectors into the HCC cell line and observed the role of SNAI1 using loss of function assays. After transfection into HepG2, SMMC‐7721 and BEL‐7402 with a MOI of 20 for 72 h, we observed the expression of EGFP using a fluorescence microscope and found that EGFP expression was highest in HepG2 (data not shown). As shown in Fig. 3D, the percentage of infection as high as 87% after lentiviral transduction. Thus, we completed the subsequent experiments using the HepG2 cell line. As shown in Fig. 3E,F,G, the expression of SNAI1 was decreased compared with that of the scrambled control LV‐RNAi after transfection for 48 and 72 h, particularly at 72 h. At the same time, we observed the expression of E‐cadherin and N‐cadherin, which are two key EMT‐related molecules, after knockdown of SNAI1. As shown in Fig. 3G, E‐cadherin expression was decreased. By contrast, N‐cadherin expression was increased. The results were consistent with those of previous reports 15. All together, these data suggest that the expression of SNAI1 is induced in HCC cell lines, and the above three lentiviral vectors expressing RNAi against SNAI1 all work to interfere with its expression, particularly LV‐SNAI1‐RNAi #3.

### SNAI1 RNAi promotes the apoptosis of HepG2 cell through reducing Bcl‐2 expression

**Figure 4:**
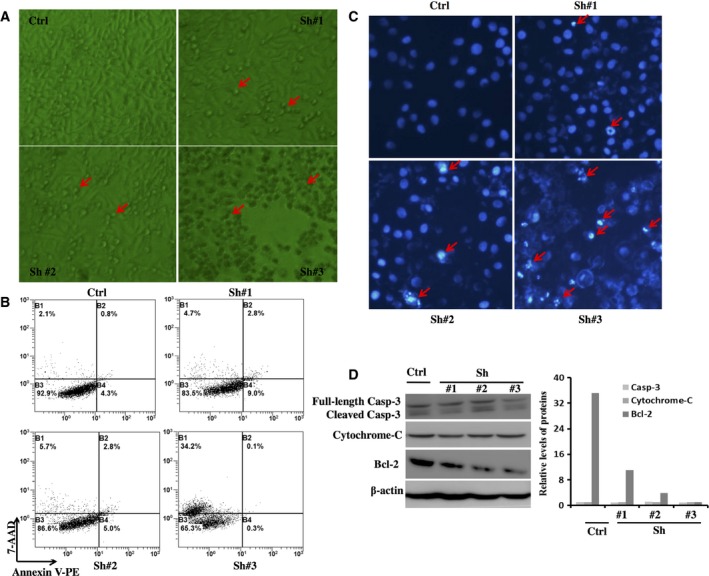
Downregulation of SNAI1 expression increases the apoptosis of HepG2 cell through reducing Bcl‐2 expression. After lentiviral vectors expressing RNAi against SNAI1 or scrambled control vectors were transfected into HepG2 cells with an MOI of 20 for 96 h, we observed the growth state of HepG2 cells under an optical microscope (A: × 40 original magnification; Red arrows, apoptotic cells), and these cells were collected and stained with 7AAD/Annexin V to analyze apoptosis by flow cytometry (B). Hoechst staining was completed (C: × 200 original magnification; Red arrows, chromatin condensation, DNA fragmentation and chromatin margination). The expression levels of Bcl‐2, Cytochrome‐C and Caspase‐3 were measured in HepG2 cells transfected with lentiviral vectors for 72 h by western blotting (D). Similar observations were obtained in three independent experiments.

To explore the possible functions of SNAI1 in HCC cell apoptosis and death, three lentiviral siRNA vectors against SNAI1 or scrambled control vectors were transfected into HepG2 cells. Surprisingly, we observed under an optical microscope that HepG2 cells underwent obvious apoptosis and death after transfection for 96 h compared with controls (Fig. 4A), particularly LV‐SNAI1‐RNAi #3. Subsequently, we collected these cells using trypsin without EDTA and stained them using the 7AAD/Annexin V kit to analyze by flow cytometry. As shown in Fig. 4B, few 7AAD‐ and Annexin V‐positive cells were detected in the scrambled control‐treated cells; however, the percentage of apoptotic and dead cells was increased in LV‐SNAI1‐RNAi #1‐, #2‐ and #3‐transfected cells. Moreover, there was dramatically increased death in LV‐SNAI1‐RNAi #3‐transfected cells. Meanwhile, we detected apoptotic cells by Hoechst staining. As shown in Fig. 4C, the percentage of apoptotic cells in the RNAi‐transfected groups was significantly increased compared with that in the control group. The nuclear edge of the control group was clear and integrated. However, apoptotic salient features such as chromatin condensation, DNA fragmentation and chromatin margination were found in LV‐SNAI1‐RNAi #1‐, #2‐ and #3‐transfected cells. In the LV‐SNAI1‐RNAi #3 group, we also observed cell disruption and chromatin release, so some cells were stained in the cytoplasm or around the cytoplasm. In addition, to determine which molecules play an important role in inducing apoptosis after the ablation of SNAI1, we detected the expression of relevant apoptotic molecules, including Bcl‐2, Cytochrome‐C and Caspase‐3, after interference of SNAI1 by western blotting. As shown in Fig. 4D, SNAI1 RNAi treatment significantly inhibited the expression of Bcl‐2. However, the expression of Cytochrome‐C and Caspase‐3 were little affected. Taken together, these results indicate that downregulating SNAI1 in HepG2 cells increased apoptosis via diminishing Bcl‐2, but not Cytochrome‐C and Caspase‐3.

### SNAI1 RNAi inhibits the growth of HepG2 cells by inducing G1 arrest and suppressing Cyclin D1 expression, restraining the migration of these cells

**Figure 5:**
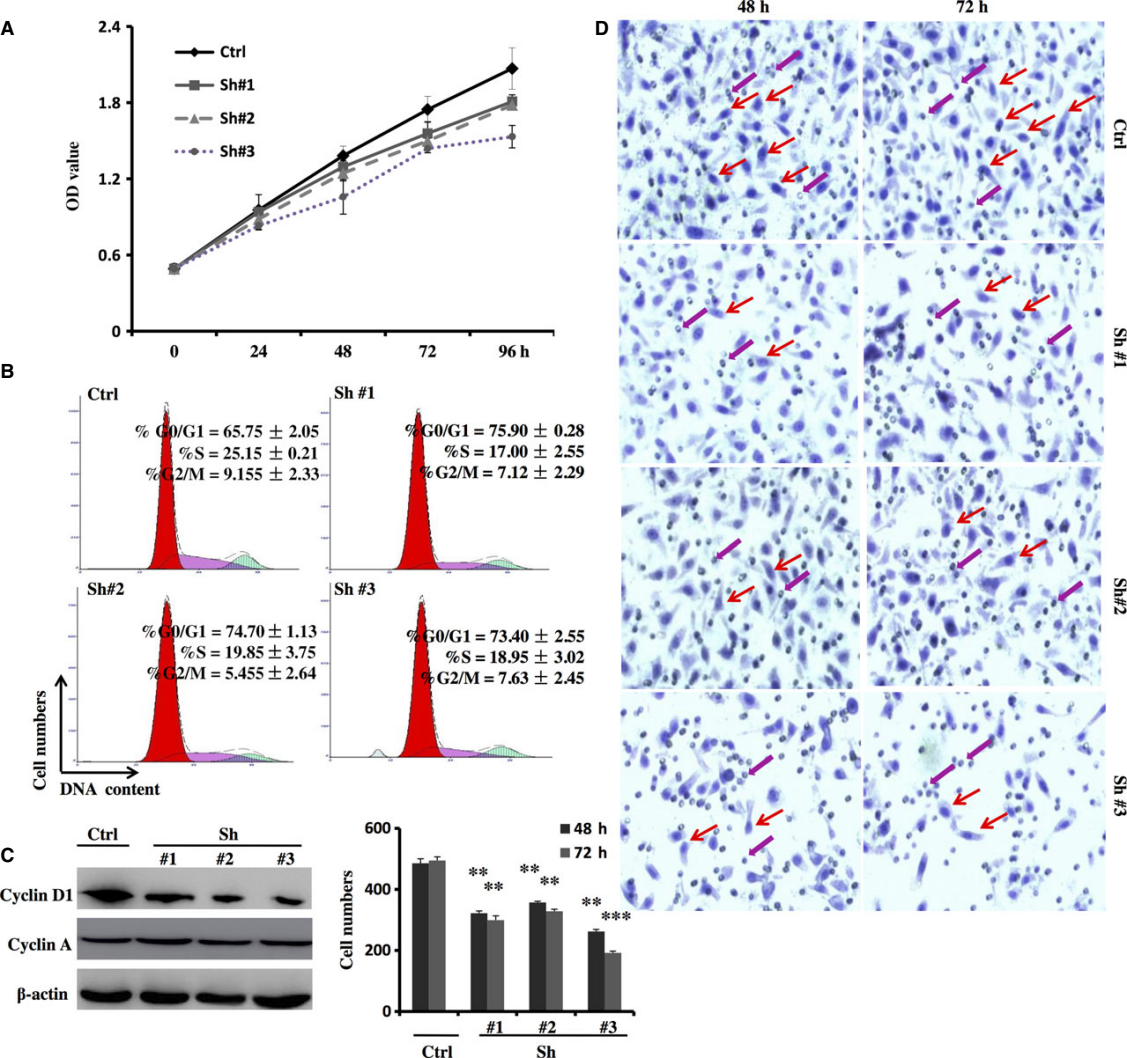
Downregulation of SNAI1 expression inhibits the growth and migration of HepG2 cells. The proliferation of HepG2 cells transfected lentiviral vectors was assayed over a 96‐h period, and the data shown are means ± SD from five independent experiments (A). HepG2 cells transfected with lentiviral vectors for 96 h were analyzed by propidium iodide staining and flow cytometry. A representative plot from one experiment and means ± SD from three independent experiments are shown (B). Cyclin D1 and Cyclin A expression was detected in HepG2 cells transfected with lentiviral vectors for 72 h by western blotting (C). HepG2 cells transfected with lentiviral vectors for 48 or 72 h were seeded to the upper chamber at a concentration of 1.5 × 10^5^ cells per well for 18 h, and migrated cells on the lower surface of the membrane were stained with hematoxylin, × 200 original magnification. A representative plot from one experiment is shown (D: Red arrows, migrated cells; Purple arrow, transwell's hole). Additionally, the average number of the cells per field was counted: × 10 objective magnification from five microscopic fields (E). **P < 0.01, ***P < 0.001.

To further investigate the effect of SNAI1 in HCC cell growth and metastasis, we also knocked down the expression of SNAI1 with the above lentiviral vectors. As an initial step, the capacity of proliferation was evaluated in HepG2 cells transfected with lentiviral vectors using the CCK8 assay. As shown in Fig. 5A, results showed that the SNAI1 knockdown groups had a much lower optical density than the scrambled control group. Next, we examined the HepG2 cell cycle after different treatments using flow cytometry. As expected, after transfection for 96 h, the LV‐SNAI1‐RNAi #1, #2 and #3 group cells were stagnated in G0/G1 phase, whereas the cell numbers of S and G2/M phase were observably declined (Fig. 5B). Notably, the LV‐SNAI1‐RNAi #3 group showed an apoptotic peak before G0/G1 phase (Fig. 5B), a finding that was consistent with the results of the 7AAD/Annexin V assay (Fig. 4B). To explore the mechanism by which SNAI1‐RNAi inhibits cell growth, we analyzed the expression of potential cell cycle regulators Cyclin D1 and Cyclin A after serum starvation. As shown in Fig. 5C, downregulation of SNAI1 by RNAi in HepG2 cells remarkably abrogated Cyclin D1 but had no effect on the Cyclin A protein level. In addition, we evaluated HepG2 cell migration via transwell assay. As shown in Fig. 5D,E, the cell numbers of migration in the LV‐SNAI1‐RNAi #1, #2, and #3 groups were decreased compared with the scrambled control group. All together, these data indicate that downregulation of SNAI1 arrests cell cycle progression by reducing Cyclin D1 but not Cyclin A. Meanwhile, SNAI1 RNAi represses the migration of HepG2 cells.

### SNAI1 RNAi represses tumor formation in BALB/c nude mice

**Figure 6:**
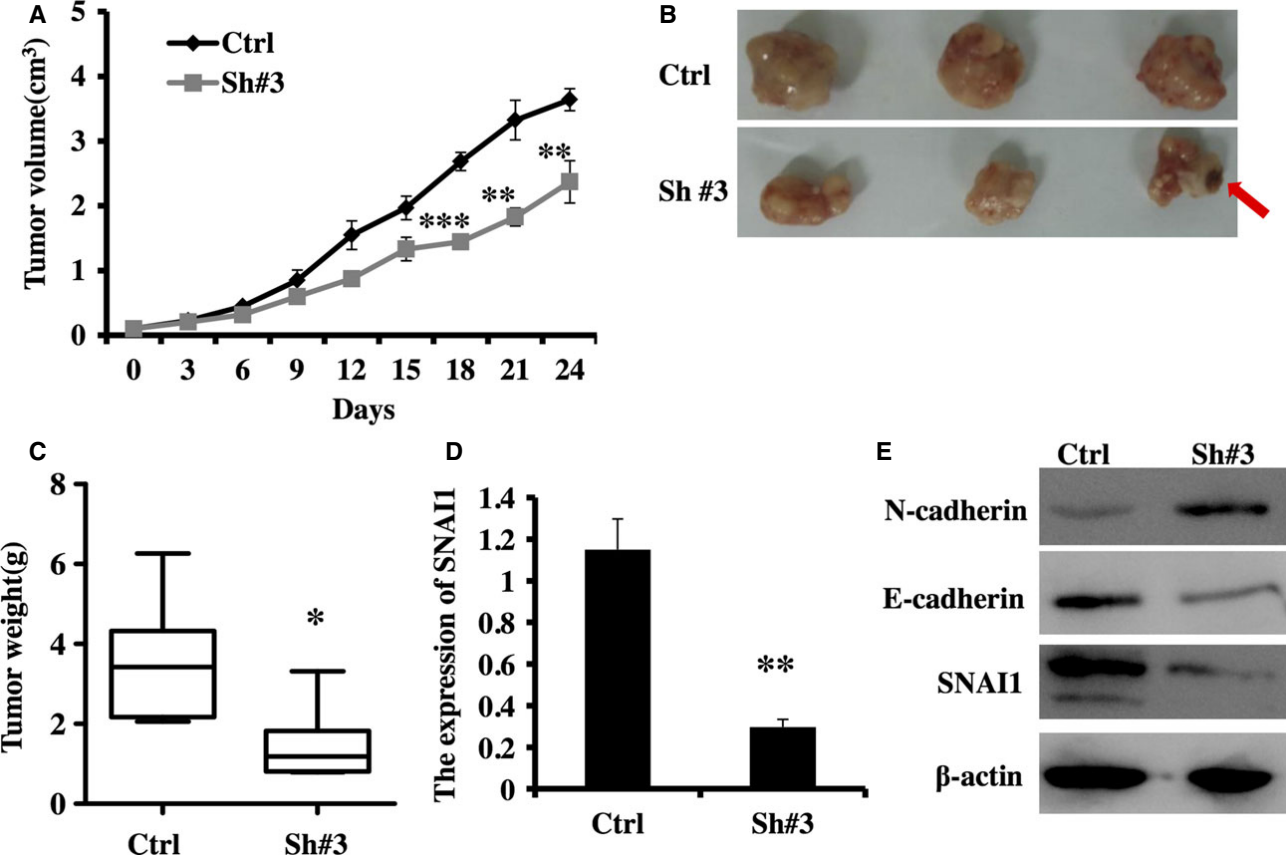
Downregulation of SNAI1 expression represses tumor formation in BALB/c nude mice. (A) Xenograft tumor growth determined over a 24‐day period (means ± SD,* n* = 6). Images of tumors injected with lentiviral vectors from each group are shown (B: Red arrows, necrotic tissue), and the weight of these tumors were measured at the time of death (mean ± SD,* n* = 6) (C). Real‐time PCR analyzed the expression of SNAI1 mRNA in tumors injected with lentiviral vectors (mean ± SD,* n* = 6) (D). Western blotting analyzed the expression of SNAI1, E‐cadherin and N‐cadherin in tumors injected with lentiviral vectors (mean ± SD,* n* = 6) (E). *P < 0.05, **P < 0.01., ***P < 0.001.

To further evaluate the influence of SNAI1 RNAi *in vivo*, nude mice were subcutaneously inoculated into HepG2 cells (1 × 10^7^). After reaching a diameter of 0.5 cm, the tumors were injected with control LV‐ RNAi or LV‐SNAI1‐RNAi #3 vectors. As shown in Fig. 6A, injection of LV‐SNAI1‐RNAi #3 vectors significantly inhibited tumor growth. Consistent with this finding, the weight of RNAi #3‐injected tumors was less than that of LV‐RNAi‐injected control tumors (Fig. 6B,C). Moreover, we observed necrotic tissue in RNAi #3‐injected tumors (Fig. 6B). After mice were sacrificed, we detected SNAI1 expression using real‐time PCR assay and found that the expression of SNAI1 was obviously reduced in RNAi #3‐injected tumors compared with that in LV‐RNAi‐injected control tumors (Fig. 6D). Meanwhile, we observed the expression of E‐cadherin and N‐cadherin using western blot assay and found that E‐cadherin expression was decreased, and N‐cadherin expression was increased in RNAi #3‐injected tumors compared with those in control‐injected tumors. The results were consistent with those in the above study using the HepG2 cell line *in vitro*. Taken together, these data show that the inhibitory effect of SNAI1 RNAi also existed *in vivo*.

## Discussion

In this report, we demonstrated that the expression of SNAI1 is increased in the HCC sample obtained from surgical resection. Moreover, the abnormally higher expression correlated with invasion, metastasis, and poor prognosis. In addition, downregulation of SNAI1 expression led to increased HepG2 cell apoptosis and decreased proliferation and migration. Similarly, the role of lentiviral‐mediated RNAi vectors against SNAI1 existed in nude mice.

Epithelial–mesenchymal transition plays a pivotal role in cancer cell metastasis via increasing cell mobility 15, 16. SNAI1 was reported to be a zinc‐finger transcriptional repressor that is associated with EMT through restraining the expression of E‐cadherin 17, 18, 19, a key regulator of cell–cell adhesion 20. Zhang *et al*. 21 showed that prostaglandin E2 upregulated SNAI1, leading to invasion. Until now, the overexpression of SNAI1 has been extensively evaluated in different human cancer tissues and cell lines, particularly colon cancer 22, gastric cancer 23, breast cancer 24, and ovarian cancer 25. Additionally, our previous study found that SNAI1 is abnormally highly expressed in human liver cancer tissues by western blotting, and SNAI1 is remarkably associated with poor prognosis and recurrence of HCC by ROC and survival analysis 10. However, there has been no further validation of how SNAI1 overexpression regulates the malignant phenotype of HCC, such as proliferation and apoptosis.

Consistent with our previous findings, we found that the expression of SNAI1 is significantly increased in 34 of 42 cases by immunohistochemistry, and the positive rate of high expression was up to 80.95% in HCC. In addition, we analyzed the correlation between the expression of SNAI1 and certain clinical parameters. These results showed that SNAI1 expression was correlated with distal metastasis, incomplete tumor capsule formation and histological differentiation. *In vitro*, the lentiviral‐mediated RNAi assay demonstrated that SNAI1 knock down effectively reduces HepG2 cell proliferation, migration and invasion and promotes HepG2 cell apoptosis. Kurrey *et al*. 26 have reported that SNAI1/2 can directly decrease the expression of proapoptotic genes such as PUMA, ATM and PTEN to resist p53‐ mediated apoptosis in ovarian cancer cells. However, in this study, we indicated that knock down of SNAI1 promoted cell apoptosis in HepG2 cells by raising antiapoptotic protein level of Bcl‐2. β‐catenin is a main downstream effector of the canonical Wnt pathway and is implicated in governing the self‐renewal of various normal and cancer stem cells 27, 28, 29. Scherbakov *et al*. have shown that breast cancer cells can resist hypoxia through the SNAI1/beta‐catenin signal pathway 30, 31. Nevertheless, in this study, we substantiated that the downregulation of SNAI1 leads to cells stagnating in the G0/G1 phase by using flow cytometry. In parallel, we observed that the knock down of SNAI1 suppresses the expression of Cyclin D1 but not that of Cyclin A. These results implied that the overexpression of SNAI1 may be responsible for the progression and poor prognosis of HCC patients. Because the current biomarkers (including AFP, CEA, GPC‐3 and CD2) have limited sensitivity and specificity, new biomarkers are needed to improve the diagnosis and management of HCC patients. Our results partially support that SNAI1 can serve as a novel biomarker to estimate the progression and poor prognosis of HCC patients.

From another perspective, regarding whether SNAI1 is a target for the biological treatment of HCC, we observed the role of lentiviral‐mediated RNAi vectors against SNAI1 using a nude mouse tumor model *in vivo*. Fortunately, our data showed that the knock down of SNAI1 can significantly diminish the tumor volume and weight and occasionally induce necrosis. This result is consistent with that obtained *in vitro*.

In summary, our study has demonstrated an important role for SNAI1 in the regulation of growth and apoptosis of HepG2 cells. Moreover, the positive role of SNAI1 in the malignant behavior of HepG2 cells was also verified in nude mice. Given the correlation with the abnormally higher expression of SNAI1 and clinical features of HCC patients, SNAI1 may be a novel biomarker and may have therapeutic potential for HCC patients.

## Author contributions

WR and CQ conceived and designed the experiments. JQ, HB, and FL analyzed the data. YJ, SG, and JS contributed reagents/materials/analysis tools. JQ, TL, and HB performed the experiments and wrote the paper.
